# Tissue-Specific Redistribution of Free Amino Acids in Mandarin Fish (*Siniperca chuatsi*) Under Acute Salinity, Alkalinity and Combined Saline–Alkaline Stress

**DOI:** 10.3390/life16061031

**Published:** 2026-06-19

**Authors:** Yan Li, Longyi Li, Yiming Li, Qiang Ji, Zongli Yao, Pengcheng Gao, Kai Zhou, Zhen Sun, Yuxing Wei, Qifang Lai

**Affiliations:** 1 East China Sea Fisheries Research Institute, Chinese Academy of Fishery Sciences, Shanghai 200090, China; ly508109@163.com (Y.L.); 18874172728@163.com (L.L.); liym@ecsf.ac.cn (Y.L.); yaozl@ecsf.ac.cn (Z.Y.); gaopc@ecsf.ac.cn (P.G.); zhoukai69@126.com (K.Z.); sunzhen@ecsf.ac.cn (Z.S.); weiyx@ecsf.ac.cn (Y.W.); 2 College of Fisheries and Life Science, Dalian Ocean University, Dalian 116023, China; 3 Shanghai Songjiang District Aquatic Technology Promotion Station, Shanghai 201799, China; cc7766@163.com

**Keywords:** *Siniperca chuatsi*, saline–alkaline stress, free amino acids, tissue specificity

## Abstract

Free amino acids (FAAs) are important low-molecular-weight metabolites involved in osmotic regulation, acid–base balance, and nitrogen metabolism in fish exposed to saline–alkaline environments. To characterize tissue-specific FAA responses in mandarin fish (*Siniperca chuatsi*), 10 cm juveniles were exposed for 96 h to freshwater control (FW), salinity stress (S, salinity 8), alkalinity stress (A, alkalinity 20 mmol/L), or combined saline–alkaline stress (SA, salinity 8 + alkalinity 20 mmol/L). The contents of 19 FAAs were compared among plasma, muscle, liver, brain, and kidney. FAA profiles showed clear tissue specificity. Total FAA (17) decreased in plasma under all stress treatments, increased in muscle under S and SA but decreased under A, increased in liver and kidney, and decreased under single stress but increased under combined stress in brain. Distinct tissue distribution patterns were observed for functional FAA groups. Under salinity stress, osmoregulation-related FAAs, particularly Ala and Pro, showed higher contents mainly in muscle, liver, and kidney. Under alkalinity stress, kidney showed concurrent increases in multiple FAAs, including Ala, Pro, Glu, Gln, Val, Ile, and Leu, whereas brain was characterized by a high Gln content. Under combined saline–alkaline stress, liver was the main tissue in which multiple functional FAA groups increased simultaneously, kidney maintained elevated levels of several FAAs, and brain showed treatment-specific high levels of Gln and Tau. Redundancy analysis (RDA) indicated weak constrained explanatory power of salinity and alkalinity for the overall FAA profile, whereas tissue-specific differentiation was evident. Glu, Gln, and Pro showed directional consistency with the salinity vector, whereas Val and Leu tended to align with the alkalinity-related ordination direction. Overall, acute saline–alkaline exposure induced a functional and tissue-specific distribution pattern of FAAs rather than a uniform whole-body shift in mandarin fish.

## 1. Introduction

Environmental salinization is an increasingly important issue affecting the utilization of aquatic and land resources, and saline–alkaline water resources may provide additional space for aquaculture expansion [1,2]. For economically important freshwater fish, the ability to maintain osmotic homeostasis, acid–base balance, and metabolic stability under changing salinity and carbonate alkalinity is central to saline–alkaline adaptation. Mandarin fish (*Siniperca chuatsi*) is an important freshwater aquaculture species in China. Recent work has shown that juvenile mandarin fish can tolerate certain low-salinity and alkaline conditions, accompanied by physiological responses related to osmoregulation and acid–base balance [3]. Increased salinity alters the osmotic gradient between fish and the external environment, thereby affecting ion transport, osmoregulation, and energy allocation. Elevated carbonate alkalinity is commonly associated with high pH, altered ionic composition, and impaired ammonia excretion, which further challenges internal homeostasis [4,5,6].

Free amino acids (FAAs) are sensitive metabolic components of fish responses to salinity and alkalinity. Some FAAs function as compatible organic osmolytes involved in cell volume regulation, whereas others participate in nitrogen metabolism and stress-related substrate mobilization [7]. Environmental salinity can rapidly alter plasma FAA levels in euryhaline fish, and Mozambique tilapia shows tissue-specific changes in organic osmolytes in brain, kidney, muscle, and liver under different salinity and temperature conditions [8,9]. In Nile tilapia, Mozambique tilapia, and other fishes, salinity, alkalinity, and combined saline–alkaline conditions can alter FAA contents and related metabolic processes in muscle, gill, and other tissues [10,11,12,13]. Thus, FAA content profiles can provide metabolic evidence for tissue-specific roles in osmoregulation, acid–base/nitrogen metabolism, and substrate mobilization under saline–alkaline stress.

Most previous studies have focused on a single tissue, a single stressor, or a limited set of metabolic indicators. Systematic evidence remains limited regarding whether different functional FAAs exhibit coordinated or tissue-specific redistribution patterns under salinity, alkalinity, and combined saline–alkaline stress in mandarin fish. Multi-tissue omics studies have shown that fish responses to salinization are strongly tissue-specific, suggesting that multi-tissue analysis can provide a more integrated view of adaptive responses than single-tissue observations [14]. In this study, plasma, muscle, liver, brain, and kidney were selected because they represent different physiological compartments. Plasma reflects circulating metabolic status, muscle is a major reservoir of free amino acids and potential organic osmolytes, liver is central to amino acid metabolism and carbon–nitrogen skeleton conversion, brain is sensitive to nitrogen-related metabolic disturbance, and kidney is involved in osmoregulatory and nitrogenous waste-related processes. In addition, total FAA content reflects the overall size of the free amino acid pool, whereas Ala, Gln, Pro, Tau, and BCAAs are representative FAA-related components commonly associated with amino acid metabolism, nitrogen-related adjustment, substrate redistribution, and osmotic responses in aquatic animals. Therefore, the present study compared the contents of 19 FAAs in plasma, muscle, liver, brain, and kidney of 10 cm mandarin fish after 96 h of acute exposure to salinity, alkalinity, or combined saline–alkaline stress, with the aim of clarifying tissue-specific FAA redistribution patterns and providing basic metabolic information for understanding the response of mandarin fish to saline–alkaline environments.

## 2. Materials and Methods

### 2.1. Experimental Fish and Acclimation Conditions

Mandarin fish (*Siniperca chuatsi*) were obtained from a mandarin fish culture base in Hefei, Anhui Province, China, and transported alive with oxygenation to the East China Sea Fisheries Research Institute, Chinese Academy of Fishery Sciences. Healthy fish with intact body surfaces, normal swimming behavior, and uniform size were selected for the experiment. Mean total length and body weight were 9.68 ± 0.43 cm and 12.53 ± 2.63 g, respectively. Before exposure, 150 fish were acclimated for more than 7 d in 1500 L white polyvinyl chloride culture tanks under normal indoor laboratory light conditions, and no total-darkness condition was applied. During acclimation, continuous aeration was provided, fish were fed twice daily, and 90% of the water was renewed each day. The acclimation water was tap water purified using a household central water purification system (AC/KDF-150C; Canature, Shanghai, China) and then fully aerated for more than 24 h before use. Water temperature was maintained at approximately 24 °C, pH was approximately 7.4, and dissolved oxygen remained above 8.0 mg/L. Residual chlorine was not measured during acclimation. Fish were fasted for 24 h before the experiment to minimize the influence of feeding on tissue FAA contents.

### 2.2. Preparation of Experimental Water and Treatment Design

All experimental water was prepared using tap water purified with the same household central water purification system and fully aerated for more than 24 h before use. Salinity was adjusted using commercial sea salt, and alkalinity was adjusted using sodium bicarbonate (NaHCO_3_). Experimental water for each treatment was prepared 1 d before exposure. The selected salinity and alkalinity levels were based on preliminary exposure tests. These levels did not cause mortality under either single-factor or combined saline–alkaline conditions during 96 h of exposure, while still producing sufficiently strong physiological responses for subsequent FAA analysis. The exposure experiment was conducted in 100 L blue polyvinyl chloride culture tanks under normal indoor laboratory light conditions, and no total-darkness condition was applied during exposure. Each treatment consisted of three independent replicate tanks, with 10 fish maintained in each tank. Therefore, 30 fish were used in each treatment group, and a total of 120 fish were used in the exposure experiment. Fish were randomly allocated to the replicate tanks before exposure, and each tank was treated as an independent experimental unit. To reduce tank-related variation, all replicate tanks were maintained under the same water volume, stocking density, aeration, indoor light condition, temperature, and daily water-renewal regime. Water quality was monitored daily throughout the exposure period. Salinity and pH were measured using a YSI multiparameter meter, whereas alkalinity was determined by hydrochloric acid titration. Dissolved oxygen was maintained above 10 mg/L by continuous aeration. The measured salinity, alkalinity, and pH values during exposure are presented in Table 1.

During the experiment, water temperature was maintained at approximately 24 °C with continuous aeration. Fish were not fed, and 90% of the water was renewed daily to reduce metabolic waste accumulation and maintain relatively stable water quality. Residual chlorine was not measured during the exposure experiment.

### 2.3. Tissue Sampling and FAA Determination

At 96 h of exposure, three fish were randomly sampled from each replicate tank in each treatment group. Thus, nine fish were sampled from each treatment group. For each replicate tank, tissues of the same type collected from the three sampled fish were pooled to generate one tank-level pooled biological replicate. Therefore, each treatment–tissue combination had three tank-level pooled biological replicates. Before sampling, fish were anesthetized with 100 mg/L of MS-222. Plasma, muscle, liver, brain, and kidney samples were collected and pooled separately by tissue type within each replicate tank. Samples were rapidly frozen in liquid nitrogen and stored at −80 °C until analysis.

FAA analysis was performed using an automatic amino acid analyzer (S433D; Sykam, Germany). The contents of 19 FAA-related components, including 17 conventional FAAs together with Gln and Tau, were determined using the external standard method. Amino acid standards were used to establish standard calibration curves. Prior to formal analysis, preliminary samples were submitted to the testing laboratory to confirm that they met the analytical requirements. Quality control was performed using quality-control samples and replicate injections according to the analytical procedures of the testing laboratory.

The original analytical results were reported as mass percentages (%), equivalent to g/100 g sample according to the analytical report. For clearer interpretation, all FAA contents were converted to µg/g sample using the conversion factor 1% = 10,000 µg/g. Total FAA (17) was calculated as the sum of the 17 conventional FAAs. Gln and Tau were not included in total FAA (17) and were analyzed separately because of their specific relevance to nitrogen metabolism and osmoregulation, respectively.

### 2.4. Statistical Analysis

Raw replicate values were converted to µg/g sample before statistical analysis. Data are presented as mean ± standard deviation (SD), with *n* = 3 tank-level pooled biological replicates for each treatment–tissue combination. For each tissue and each FAA-related component, a two-way analysis of variance (ANOVA) was performed to test the main effects of salinity, alkalinity, and their interaction. The full two-way ANOVA outputs, including degrees of freedom, F-values, and *p*-values for the main effects of salinity and alkalinity and their interaction, are provided in Supplementary Tables S1–S5. When significant main effects or interactions were detected, Tukey’s post hoc test was used for multiple comparisons among the four treatment combinations. Normality and homogeneity of variance were assessed before ANOVA. For tissue distribution figures of representative FAA-related components, differences among tissues within the same treatment group were analyzed using one-way ANOVA followed by Tukey’s post hoc test (*p* < 0.05). Before RDA, FAA data were Hellinger-transformed to reduce the influence of high-abundance variables and differences in total FAA levels among tissues and to make the data more suitable for Euclidean distance-based ordination. RDA was performed using the 19 individual FAAs as response variables and salinity and alkalinity as explanatory variables, in order to visualize directional relationships between the FAA profile and environmental factors [15]. Data were organized in Excel 2021, and statistical analyses were conducted using SPSS 26.0 and R software (version 4.5.3; R Core Team, Vienna, Austria).

## 3. Results

### 3.1. Changes in FAA Contents in Plasma

Plasma FAA contents are shown in Table 2. Compared with FW, total FAA (17) decreased significantly in the S, A, and SA groups (*p* < 0.05), reaching 238.4, 228.3, and 187.6 µg/g sample, respectively, with the lowest value in the SA group. Under salinity stress, Tau, Ala, and Pro decreased simultaneously, and Val, Ile, Leu, and Lys were also significantly lower than those in FW (*p* < 0.05). Under alkalinity stress, Ala, Gly, and several neutral amino acids continued to decrease; however, under combined saline–alkaline stress, Tau, Ala, Gly, and Pro decreased further, and Val, Ile, Leu, Lys, and His were also significantly lower than those in FW. These results indicate that plasma FAA contents were mainly characterized by decreases under the three stress treatments, with the strongest overall reduction in the SA group.

### 3.2. Changes in FAA Contents in Muscle

Muscle FAA contents are shown in Table 3. Total FAA (17) changed from 2437.0 µg/g sample in FW to 2874.6, 2018.3, and 3750.9 µg/g sample in the S, A, and SA groups, respectively, indicating increases in the S and SA groups and a decrease in the A group (*p* < 0.05). The S group was mainly characterized by increases in Ala, Pro, Ser, Thr, and Glu. In the A group, Ala, Pro, Ser, Thr, and His were generally lower than those in FW, and muscle total FAA (17) also decreased, whereas Tau remained relatively high. The SA group showed broad increases in Asp, Thr, Ser, Glu, Gly, Ala, Lys, His, Arg, and Pro (*p* < 0.05). Overall, muscle responses were more closely associated with osmoregulation-related and nitrogen metabolism-related FAAs, whereas Val, Ile, and Leu did not show a generalized increase.

### 3.3. Changes in FAA Contents in Liver

Liver FAA contents are shown in Table 4. Overall, FAA contents increased in liver under all three stress treatments. Total FAA (17) increased from 25,054.8 µg/g sample in FW to 38,632.3, 27,769.4, and 44,524.7 µg/g sample in the S, A, and SA groups, respectively, with the highest value in the SA group (*p* < 0.05). Most conventional FAAs increased markedly in the S and SA groups, particularly Glu, Ala, Gly, Pro, Val, Ile, and Leu. Although the magnitude of increase in the A group was lower than that in the S and SA groups, several FAAs still increased relative to FW. Overall, liver showed elevated levels of osmoregulation-related FAAs, acid–base/nitrogen metabolism-related FAAs, and BCAAs in the S and SA groups, and was the main tissue showing simultaneous increases in multiple functional FAA groups under combined stress.

### 3.4. Changes in FAA Contents in Brain

Brain FAA contents are shown in Table 5. Brain total FAA (17) changed from 9348.7 µg/g sample in FW to 7968.3, 7621.2, and 9891.9 µg/g sample in the S, A, and SA groups, respectively. Compared with FW, brain total FAA (17) decreased significantly in the S and A groups but increased significantly in the SA group (*p* < 0.05). In the S and A groups, Ala, Gly, Pro, and several neutral FAAs decreased markedly. In the SA group, Tau, Gly, Ala, Pro, and Gln were higher than those under single-stress conditions. The most prominent features of brain tissue were the high Gln level in the A and SA groups and the increase in Tau in the SA group, suggesting treatment-specific FAA distribution patterns related to nitrogen metabolism and local homeostasis.

### 3.5. Changes in FAA Contents in Kidney

Kidney FAA contents are shown in Table 6. Total FAA (17) increased from 27,124.4 µg/g sample in FW to 29,683.2, 39,689.4, and 34,853.6 µg/g sample in the S, A, and SA groups, respectively; all stress treatments were higher than FW, with the highest value in the A group (*p* < 0.05). Under salinity stress, Tau, Ala, Gly, Pro, Val, Ile, and Leu increased together, whereas Lys decreased markedly. Under alkalinity stress, Ala, Gly, Pro, Val, Leu, Phe, Tyr, His, and Arg were higher than in FW, and Gln and Glu also remained at relatively high levels. Under combined saline–alkaline stress, Ala, Gly, Pro, Tau, and several neutral amino acids remained high, although the overall magnitude was slightly lower than that in the A group. Overall, kidney showed simultaneous increases in total FAA (17), Ala, Pro, Glu, Gln, Val, Ile, and Leu in the A group, making it the main tissue with elevated contents of multiple FAAs under alkalinity stress.

### 3.6. Tissue Distribution Patterns of Representative FAAs and RDA

**Figure 1:**
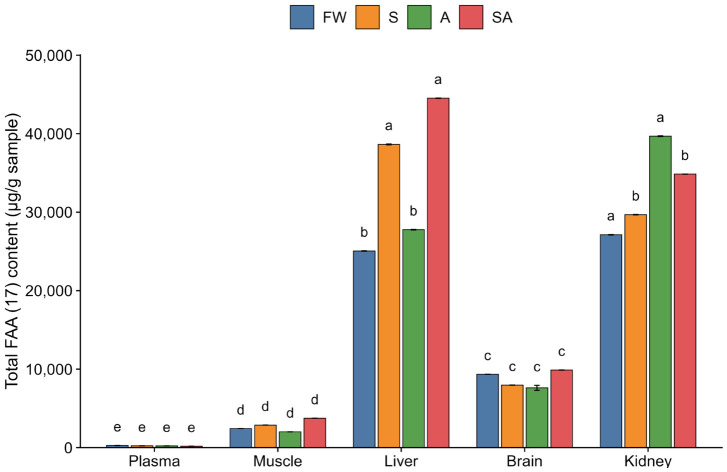
Total free amino acid content [Total FAA (17)] in plasma, muscle, liver, brain, and kidney of mandarin fish after 96 h exposure to freshwater control (FW), salinity stress (S), alkalinity stress (A), and combined saline–alkaline stress (SA). Values are expressed as mean ± SD (*n* = 3 tank-level pooled biological replicates) in µg/g sample. Different lowercase letters indicate significant differences among tissues within the same treatment group (*p* < 0.05).

**Figure 2:**
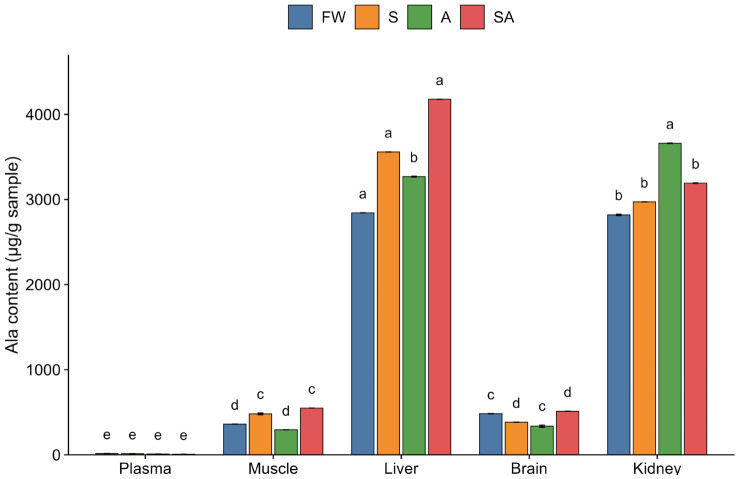
Glutamine (Gln) content in plasma, muscle, liver, brain, and kidney of mandarin fish after 96 h exposure to freshwater control (FW), salinity stress (S), alkalinity stress (A), and combined saline–alkaline stress (SA). Values are expressed as mean ± SD (*n* = 3 tank-level pooled biological replicates) in µg/g sample. Different lowercase letters indicate significant differences among tissues within the same treatment group (*p* < 0.05).

**Figure 3:**
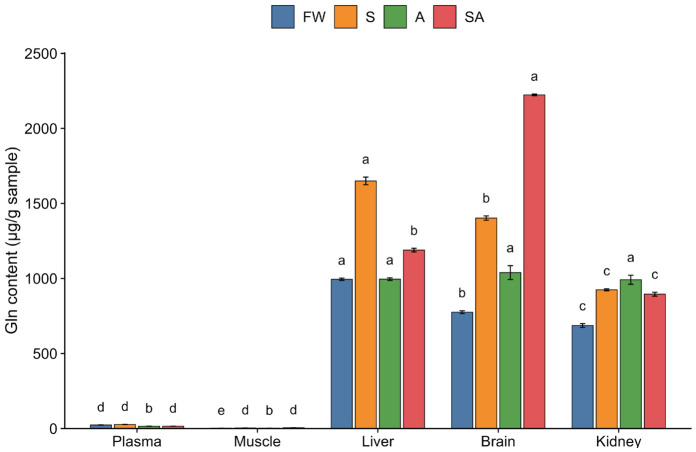
Alanine (Ala) content in plasma, muscle, liver, brain, and kidney of mandarin fish after 96 h exposure to freshwater control (FW), salinity stress (S), alkalinity stress (A), and combined saline–alkaline stress (SA). Values are expressed as mean ± SD (*n* = 3 tank-level pooled biological replicates) in µg/g sample. Different lowercase letters indicate significant differences among tissues within the same treatment group (*p* < 0.05).

**Figure 4:**
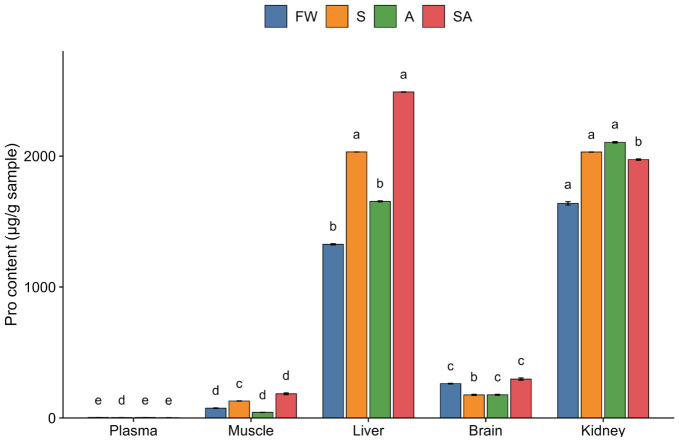
Proline (Pro) content in plasma, muscle, liver, brain, and kidney of mandarin fish after 96 h exposure to freshwater control (FW), salinity stress (S), alkalinity stress (A), and combined saline–alkaline stress (SA). Values are expressed as mean ± SD (*n* = 3 tank-level pooled biological replicates) in µg/g sample. Different lowercase letters indicate significant differences among tissues within the same treatment group (*p* < 0.05).

**Figure 5:**
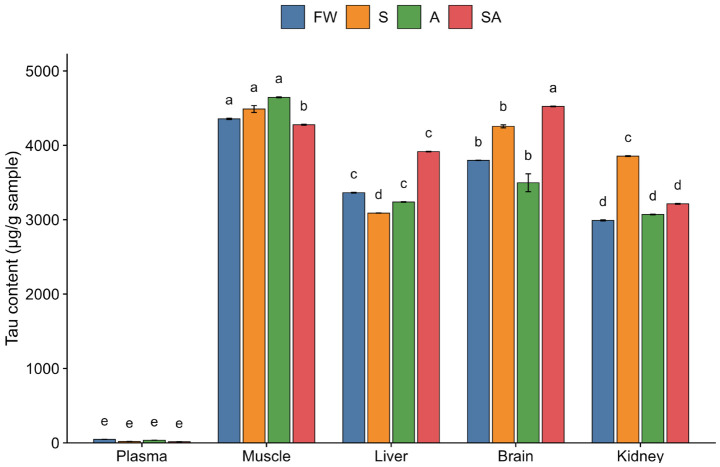
Taurine (Tau) content in plasma, muscle, liver, brain, and kidney of mandarin fish after 96 h exposure to freshwater control (FW), salinity stress (S), alkalinity stress (A), and combined saline–alkaline stress (SA). Values are expressed as mean ± SD (*n* = 3 tank-level pooled biological replicates) in µg/g sample. Different lowercase letters indicate significant differences among tissues within the same treatment group (*p* < 0.05).

To further characterize the tissue distribution of representative FAAs, distribution graphs were generated for total FAA (17), Ala, Gln, Pro, and Tau (Figure 1, Figure 2, Figure 3, Figure 4 and Figure 5). Differences among tissues within each treatment were determined using Tukey’s post hoc test (*p* < 0.05).

Total FAA (17) showed clear tissue distribution differences within each treatment. In the FW and A groups, kidney showed the highest total FAA (17), whereas in the S and SA groups, liver showed the highest total FAA (17). Plasma remained at the lowest level across all four treatments.

Ala showed treatment-dependent tissue distribution patterns. In FW, Ala was highest in liver, followed by kidney, with lower levels in brain and muscle and the lowest level in plasma. Similar patterns were observed in the S and SA groups, whereas in the A group, the highest Ala content shifted to kidney. Overall, liver showed the highest Ala content in the FW, S, and SA groups, whereas kidney showed the highest Ala content in the A group.

Gln showed pronounced tissue differences. In the FW and S groups, Gln was highest in liver, followed by brain and kidney, while plasma and muscle remained relatively low. In the A group, the highest Gln content was observed in brain, with similar levels in liver and kidney. In the SA group, brain Gln increased further and ranked first among tissues. Overall, liver showed the highest Gln content in the FW and S groups, whereas brain showed the highest Gln content in the A and SA groups.

The tissue distribution of Pro was similar to that of total FAA (17). In FW, Pro was highest in kidney, followed by liver, with lower levels in brain and muscle and the lowest level in plasma. In the S group, liver and kidney showed similarly high levels. In the A group, kidney remained the tissue with the highest Pro content, followed by liver. In the SA group, the highest value shifted to liver. Overall, kidney showed the highest Pro content in FW and A, liver and kidney showed similarly high levels in S, and liver showed the highest Pro content in SA.

Tau showed a distribution pattern distinct from those of the above FAAs. In the FW, S, and A groups, Tau was highest in muscle and second highest in brain, whereas liver and kidney were relatively low and plasma remained the lowest. In the SA group, the highest Tau content shifted to brain, followed by muscle. Overall, muscle showed the highest Tau content in FW, S, and A, whereas brain showed the highest Tau content in SA.

**Figure 6:**
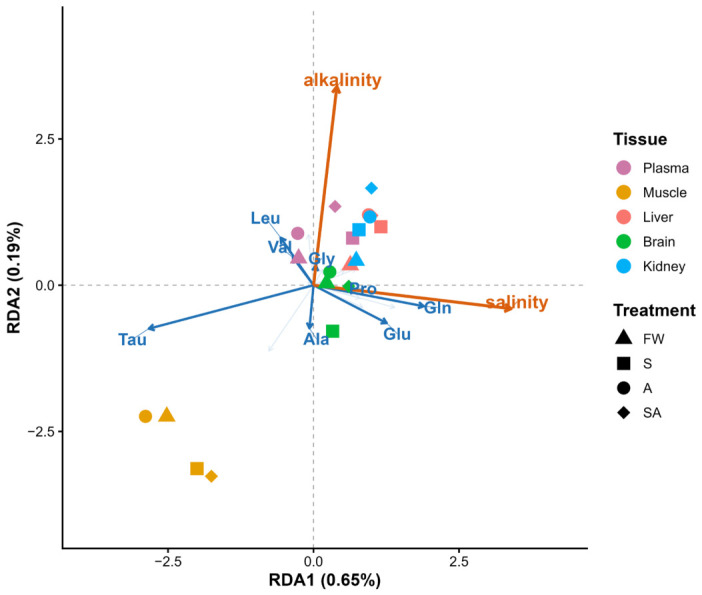
Redundancy analysis (RDA) showing the relationships between salinity, alkalinity, and free amino acid contents in plasma, muscle, liver, brain, and kidney of mandarin fish at 96 h. All 19 individual free amino acids were included in the model, whereas labels are shown only for selected representative FAAs to improve readability. Points represent the centroids of tissue × treatment combinations. Dashed lines indicate the zero reference lines for the two ordination axes.

The RDA results (Figure 6) showed that salinity and alkalinity had low constrained explanatory power for the overall profile of 19 individual FAAs. RDA1 and RDA2 explained 0.65% and 0.19% of the total variation, respectively, indicating that the direct explanatory ability of these two environmental factors was limited. In the ordination plot, separation among tissues was more evident than separation among treatments. Muscle samples were mainly located in the lower-left region and were clearly separated from other tissues, whereas plasma, liver, and kidney samples were mostly distributed from the upper to the upper-right regions with partial overlap. Brain samples were located close to the origin.

This suggests that FAA content changes associated with the two factors were not identical in ordination space. Among representative FAAs, Glu, Gln, and Pro showed directional consistency with the salinity vector. Val and Leu aligned to some extent with the alkalinity-related ordination direction. Tau was located close to muscle samples, suggesting a contribution to the separation of muscle from other tissues. Gly was close to the origin, indicating a weak directional contribution to ordination separation. Given the low constrained variation explained by the RDA axes, these relationships should be interpreted as auxiliary directional evidence rather than evidence of deterministic regulation of specific FAAs by environmental factors. Overall, RDA further indicated that differences in FAA profiles were mainly characterized by tissue-specific differentiation.

## 4. Discussion

### 4.1. Osmoregulation-Related FAAs Mainly Responded in Muscle, Liver, and Kidney

Fish exposed to salinity changes must coordinate ion transport, water regulation, and cell volume maintenance. This process depends on epithelial ion regulation, particularly in the gill, as well as on intracellular organic osmolytes that help stabilize cell volume [4,5,6,7]. As compatible organic osmolytes, FAAs can contribute to osmotic homeostasis without substantially disrupting protein structure or cellular function [7]. In the present study, osmoregulation-related FAAs (Tau, Ala, Gly, and Pro) did not increase uniformly across tissues, but instead showed distinct tissue-level distribution patterns. In plasma, Tau, Ala, and Pro generally decreased in the S, A, or SA groups, indicating that the circulation was not the main compartment for elevated levels of these FAAs. In contrast, Ala and Pro increased more clearly in muscle, liver, and kidney. This pattern is consistent with local tissue osmoregulation and increased energetic demand during salinity adaptation in fish [16,17,18].

The distribution pattern of Tau differed from those of Ala and Pro. Tau was highest in muscle in the FW, S, and A groups, but the highest Tau content shifted to brain in the SA group, suggesting stronger muscle and brain specificity. Previous studies have shown that Tau contributes to salinity adaptation and cellular osmotic homeostasis in fish [19,20]. Together with the present results, the high levels of Tau in muscle and brain may reflect a demand for cell volume-stabilizing FAAs in these tissues.

The distributions of Ala and Pro were more closely associated with liver and kidney responses. Ala was highest in liver in the FW, S, and SA groups, but shifted to kidney in the A group. Pro was highest in kidney in the FW and A groups, shifted to liver in the SA group, and showed similarly high levels in liver and kidney in the S group. Previous studies in Nile tilapia have shown that salinity and alkalinity can alter muscle FAAs and related metabolism [10], and Pro metabolism is essential for alkaline adaptation [21]. Therefore, Ala and Pro responses appeared to occur mainly in tissue compartments such as muscle, liver, and kidney rather than as a uniform increase in plasma.

### 4.2. Acid–Base/Nitrogen Metabolism-Related FAAs Were Mainly Concentrated in Liver, Kidney, and Brain

Glu and Gln are key FAAs connecting amino acid transamination, nitrogen-containing substrate transfer, and ammonia buffering. Studies of fish nitrogen metabolism have shown that ammonia is a major nitrogenous metabolic product in fish, and excess ammonia can cause substantial toxicity. Some fishes alleviate ammonia toxicity by promoting ammonia excretion, incorporating ammonia into Gln, or converting nitrogenous waste into urea [22,23,24,25]. Therefore, under saline–alkaline stress, particularly when increased alkalinity may affect ammonia excretion, the tissue distribution of Glu and Gln has important interpretive value.

In the present study, Glu remained high in liver and kidney and showed clear tissue-level increases under S, A, and SA treatments. Liver is important for amino acid transamination, carbon–nitrogen skeleton conversion, and energy metabolism, whereas kidney is closely associated with osmoregulation, ion balance, and the handling of nitrogenous metabolites. The increase in Glu in liver and kidney suggests that these two tissues may have been important sites of Glu-related metabolic responses. However, because the total variation explained by the RDA was low, the directional consistency between Glu and the salinity vector should be regarded only as auxiliary evidence.

Compared with Glu, Gln showed stronger treatment specificity. In the FW and S groups, Gln was highest in liver, whereas in the A and SA groups, brain showed the highest Gln content. Fish ammonia toxicity studies indicate that the brain is sensitive to ammonia, and Gln synthesis is closely associated with brain ammonia buffering [26,27]. Therefore, the high Gln content in brain in the A and SA groups may reflect a tissue-specific feature of Gln-related nitrogen metabolism and could be associated with ammonia-buffering processes reported in previous studies, although ammonia levels were not measured in the present study.

### 4.3. Branched-Chain Amino Acids Mainly Showed High Levels in Liver and Kidney

Changes in salinity and alkalinity commonly increase the energy required to maintain ion balance, acid–base balance, and osmotic homeostasis in fish [16]. Val, Ile, and Leu are BCAAs. Like many other amino acids, BCAAs can provide carbon skeletons through catabolism and are associated with central carbon metabolism and metabolic substrate mobilization [28,29,30,31,32]. Therefore, this study did not classify Val, Ile, and Leu simply as energy-supplying amino acids, but discussed them as representative FAAs reflecting carbon skeleton supply and metabolic substrate redistribution.

In this study, Val, Ile, and Leu generally decreased in plasma but increased markedly in liver and kidney. Liver showed simultaneous increases in Val, Ile, and Leu in the S and SA groups, whereas kidney maintained high levels in the A and SA groups. In contrast, muscle did not show increases in Val, Ile, and Leu consistent with those in liver and kidney, indicating that muscle was not the main tissue with elevated BCAA contents in this study.

Because metabolic flux was not measured, the oxidative utilization or specific metabolic fate of BCAAs cannot be directly determined. Nevertheless, liver and kidney can be considered the main tissues showing elevated BCAA contents under acute saline–alkaline stress.

### 4.4. Functional FAAs Formed a Tissue-Division Redistribution Pattern Under Combined Saline–Alkaline Stress

Taken together, the tabulated results, representative FAA distribution graphs, and RDA indicate that FAA responses in mandarin fish under acute saline–alkaline stress were not confined to a single tissue or functional category. Instead, different functional FAAs showed redistribution-like patterns among multiple tissues [33,34,35]. Plasma generally showed decreases in FAAs under the three stress treatments, indicating that it was not the main compartment with elevated FAA levels. Muscle was characterized mainly by high levels of Tau and selected osmoregulation-related FAAs. Liver showed relatively high levels of Ala, Pro, Glu, Gln, Val, Ile, and Leu, whereas kidney showed concentrated increases in total FAA (17), Ala, Pro, Glu, Gln, and BCAAs in the A group. Brain showed clear treatment specificity in the distribution of Gln and Tau.

The RDA results provide auxiliary directional evidence for this tissue-division pattern. Therefore, tissue identity appeared to contribute more strongly to FAA variation than the environmental variables included in the present study. Salinity and alkalinity may have influenced FAA redistribution patterns, but their effects should be interpreted within the context of the inherent biochemical differences among tissues. Tissue separation was more evident than treatment separation, and muscle samples were clearly separated from other tissues, suggesting a relatively distinct FAA profile. Plasma, liver, and kidney shared some directional similarity in the overall FAA profile. Glu, Gln, and Pro showed directional consistency with the salinity vector, whereas Val and Leu showed some consistency with the alkalinity-related ordination direction. The different orientations of the salinity and alkalinity vectors further indicate that FAA response patterns associated with these two environmental factors were not identical in ordination space, suggesting that salinity- and alkalinity-related FAA adjustments may involve partly distinct physiological processes. Because the constrained variation explained by RDA was low, these patterns should be interpreted as directional evidence rather than proof of direct environmental regulation.

Specifically, FAAs commonly associated with osmoregulation mainly accumulated in muscle, liver, and kidney; FAAs commonly associated with acid–base and nitrogen metabolism were mainly concentrated in liver, kidney, and brain; and BCAAs such as Val, Ile, and Leu showed high levels mainly in liver and kidney. Under combined saline–alkaline stress, this tissue-division pattern was characterized by simultaneous increases in multiple FAAs in liver, sustained FAA responses in kidney, and treatment-specific Gln/Tau distribution in brain.

## 5. Conclusions

This study demonstrates that acute saline, alkalinity, and combined saline–alkaline stress induce tissue-specific FAA redistribution in juvenile mandarin fish. Salinity stress mainly affected muscle, liver, and kidney, whereas alkalinity stress caused FAA accumulation in kidney and elevated Gln in brain. Combined stress produced coordinated FAA responses among liver, kidney, and brain. These results provide multi-tissue evidence for compartmentalized FAA responses under different environmental stresses. The observed FAA patterns were strongly tissue-dependent, suggesting that tissue-specific characteristics contributed substantially to the overall variation in FAA profiles.

The main contribution of this study is highlighting tissue-specific FAA redistribution patterns. However, ion concentrations, osmolality, ammonia levels, enzyme activities, and gene expression were not directly measured, so proposed physiological implications should be regarded as plausible hypotheses rather than direct mechanistic evidence. From a practical perspective, the tissue-specific FAA profiles may serve as indicators to evaluate metabolic responses of mandarin fish under saline–alkaline aquaculture, guiding future monitoring and management strategies.

## Figures and Tables

**Table 1:** Measured water quality parameters during the 96 h exposure experiment.

Treatment	Salinity	Alkalinity (mmol/L)	pH
FW	0.02 ± 0.01	1.82 ± 0.01	7.51 ± 0.03
S	7.99 ± 0.10	1.83 ± 0.05	8.02 ± 0.03
A	0.88 ± 0.05	20.3 ± 0.76	8.25 ± 0.13
SA	8.01 ± 0.08	20.0 ± 0.33	8.31 ± 0.09

**Note:** Values are presented as mean ± SD. FW, freshwater control; S, salinity stress; A, alkalinity stress; SA, combined saline–alkaline stress.

**Table 2:** Free amino acid contents in plasma of 10 cm mandarin fish at 96 h under different treatments (µg/g sample).

Amino Acid	FW	S	A	SA
Asp	4.2 ± 0.1 ^a^	3.5 ± 0.1 ^b^	3.0 ± 0.1 ^c^	1.5 ± 0.0 ^d^
Thr	17.1 ± 0.2 ^b^	20.1 ± 0.2 ^a^	15.8 ± 0.4 ^c^	12.8 ± 0.3 ^d^
Ser	10.9 ± 0.1 ^b^	12.0 ± 0.1 ^a^	8.4 ± 0.2 ^c^	7.7 ± 0.1 ^d^
Glu	5.3 ± 0.2 ^a^	5.5 ± 0.2 ^a^	3.9 ± 0.1 ^b^	4.0 ± 0.1 ^b^
Gly	11.7 ± 0.0 ^b^	12.8 ± 0.1 ^a^	9.9 ± 0.1 ^c^	9.0 ± 0.1 ^d^
Ala	15.9 ± 0.1 ^a^	14.0 ± 0.1 ^b^	9.9 ± 0.1 ^c^	7.3 ± 0.2 ^d^
Cystine	0.2 ± 0.0 ^a^	0.1 ± 0.0 ^b^	0.1 ± 0.0 ^c^	0.1 ± 0.0 ^d^
Val	37.7 ± 0.1 ^a^	29.2 ± 0.4 ^c^	31.7 ± 0.2 ^b^	26.5 ± 0.6 ^d^
Met	9.7 ± 0.1 ^a^	9.8 ± 0.2 ^a^	8.1 ± 0.1 ^b^	7.9 ± 0.1 ^b^
Ile	25.8 ± 0.1 ^a^	18.8 ± 0.1 ^c^	20.3 ± 0.1 ^b^	17.0 ± 0.2 ^d^
Leu	43.2 ± 0.1 ^a^	32.0 ± 0.1 ^c^	34.4 ± 0.1 ^b^	28.3 ± 0.2 ^d^
Tyr	6.1 ± 0.1 ^b^	6.7 ± 0.1 ^a^	4.2 ± 0.1 ^c^	6.7 ± 0.2 ^a^
Phe	10.5 ± 0.4 ^b^	12.6 ± 0.8 ^a^	13.6 ± 0.4 ^a^	10.3 ± 0.3 ^b^
Lys	50.1 ± 0.0 ^a^	39.1 ± 0.2 ^c^	41.5 ± 0.1 ^b^	31.4 ± 0.2 ^d^
His	9.7 ± 0.3 ^a^	8.6 ± 0.3 ^b^	7.4 ± 0.1 ^c^	5.9 ± 0.1 ^d^
Arg	12.5 ± 0.4 ^a^	10.2 ± 0.2 ^b^	12.0 ± 0.2 ^a^	8.7 ± 0.3 ^c^
Pro	4.2 ± 0.1 ^a^	3.3 ± 0.1 ^b^	4.1 ± 0.1 ^a^	2.5 ± 0.1 ^c^
Total FAA (17)	274.7 ± 0.4 ^a^	238.4 ± 1.3 ^b^	228.3 ± 0.1 ^c^	187.6 ± 0.9 ^d^
Gln	23.5 ± 0.5 ^b^	27.5 ± 0.7 ^a^	15.7 ± 0.5 ^c^	16.0 ± 0.1 ^c^
Tau	47.8 ± 0.1 ^a^	19.2 ± 0.2 ^c^	35.8 ± 0.9 ^b^	15.9 ± 0.1 ^d^

**Note:** Values are presented as mean ± SD (*n* = 3 tank-level pooled biological replicates). Different lowercase letters within a row indicate significant differences among the four treatment combinations based on Tukey’s post hoc test following two-way ANOVA (*p* < 0.05). FW, freshwater control; S, salinity stress; A, alkalinity stress; SA, combined saline–alkaline stress. The full two-way ANOVA outputs for plasma FAA contents are provided in Supplementary Table S1.

**Table 3:** Free amino acid contents in muscle of 10 cm mandarin fish at 96 h under different treatments (µg/g sample).

Amino Acid	FW	S	A	SA
Asp	242.6 ± 0.6 b	171.7 ± 1.2 c	180.3 ± 8.0 c	347.9 ± 0.4 a
Thr	147.8 ± 1.9 c	261.1 ± 0.8 a	96.4 ± 1.7 d	248.4 ± 1.5 b
Ser	142.0 ± 1.8 c	338.9 ± 1.2 a	88.8 ± 2.0 d	323.0 ± 1.5 b
Glu	249.8 ± 0.8 c	417.2 ± 1.5 b	222.5 ± 1.1 d	575.1 ± 2.4 a
Gly	697.2 ± 0.9 b	567.4 ± 4.3 d	612.0 ± 0.6 c	805.5 ± 1.2 a
Ala	361.2 ± 0.5 c	480.7 ± 9.7 b	293.6 ± 0.2 d	549.4 ± 0.9 a
Cystine	2.5 ± 0.1 b	3.7 ± 0.2 a	2.5 ± 0.1 b	1.4 ± 0.0 c
Val	55.8 ± 1.3 a	39.6 ± 0.7 c	50.9 ± 1.5 b	33.9 ± 0.7 d
Met	29.3 ± 1.0 ab	27.9 ± 0.8 b	31.1 ± 1.0 a	30.3 ± 1.0 ab
Ile	45.6 ± 0.5 a	27.7 ± 0.3 c	46.0 ± 0.1 a	28.7 ± 0.1 b
Leu	66.1 ± 0.6 a	38.9 ± 1.0 c	65.3 ± 0.5 a	41.6 ± 1.1 b
Tyr	43.4 ± 0.3 a	42.0 ± 0.9 a	41.0 ± 1.3 a	36.5 ± 1.3 b
Phe	38.7 ± 0.4 b	35.3 ± 1.4 c	34.3 ± 1.0 c	41.8 ± 0.9 a
Lys	103.6 ± 1.0 d	108.1 ± 0.6 c	114.3 ± 0.2 b	267.1 ± 1.9 a
His	114.5 ± 1.0 c	167.4 ± 0.9 b	74.4 ± 0.6 d	205.1 ± 3.1 a
Arg	21.4 ± 0.6 b	17.0 ± 0.5 c	21.6 ± 1.1 b	30.2 ± 1.0 a
Pro	75.5 ± 2.4 c	130.1 ± 1.5 b	43.4 ± 0.8 d	185.0 ± 7.0 a
Total FAA (17)	2437.0 ± 4.4 c	2874.6 ± 13.5 b	2018.3 ± 11.9 d	3750.9 ± 4.1 a
Gln	2.1 ± 0.1 c	4.0 ± 0.1 b	2.2 ± 0.1 c	5.6 ± 0.1 a
Tau	4355.6 ± 9.3 c	4488.2 ± 46.0 b	4645.1 ± 6.5 a	4276.7 ± 5.9 d

**Note:** Values are presented as mean ± SD (*n* = 3 tank-level pooled biological replicates). Different lowercase letters within a row indicate significant differences among the four treatment combinations based on Tukey’s post hoc test following two-way ANOVA (*p* < 0.05). FW, freshwater control; S, salinity stress; A, alkalinity stress; SA, combined saline–alkaline stress. The full two-way ANOVA outputs for muscle FAA contents are provided in Supplementary Table S2.

**Table 4:** Free amino acid contents in liver of 10 cm mandarin fish at 96 h under different treatments (µg/g sample).

Amino Acid	FW	S	A	SA
Asp	1673.8 ± 5.3 d	3047.3 ± 5.0 b	2184.9 ± 7.8 c	3752.1 ± 2.0 a
Thr	1524.8 ± 3.9 d	2235.7 ± 2.1 b	1781.5 ± 2.6 c	2550.1 ± 0.9 a
Ser	1552.2 ± 2.1 d	2355.0 ± 1.2 b	1983.5 ± 1.4 c	2847.0 ± 2.5 a
Glu	2540.8 ± 3.2 d	4489.2 ± 3.4 b	3292.8 ± 4.6 c	5545.2 ± 13.8 a
Gly	1061.7 ± 0.4 d	1570.9 ± 1.9 b	1282.6 ± 2.0 c	1786.3 ± 0.7 a
Ala	2843.3 ± 1.4 d	3558.1 ± 0.7 b	3267.9 ± 4.9 c	4177.7 ± 1.0 a
Cystine	17.7 ± 0.6 a	13.5 ± 0.2 b	13.0 ± 0.3 b	3.0 ± 0.1 c
Val	1709.6 ± 5.9 d	2653.3 ± 2.3 b	2027.9 ± 1.3 c	2995.0 ± 2.3 a
Met	899.3 ± 0.8 d	1329.0 ± 0.5 b	970.8 ± 0.5 c	1530.5 ± 0.6 a
Ile	1358.0 ± 1.8 d	2238.9 ± 2.7 b	1536.4 ± 2.9 c	2536.1 ± 1.0 a
Leu	2533.8 ± 2.6 d	3944.3 ± 3.2 b	2883.9 ± 2.4 c	4731.5 ± 2.2 a
Tyr	973.2 ± 2.4 d	1777.8 ± 5.7 b	1137.0 ± 4.5 c	1943.5 ± 6.4 a
Phe	1582.8 ± 23.1 d	2519.3 ± 2.6 b	1992.3 ± 1.3 c	3222.6 ± 13.6 a
Lys	1792.0 ± 3.6 b	2096.4 ± 31.9 a	478.0 ± 4.3 c	1824.6 ± 2.5 b
His	640.5 ± 4.7 c	1083.1 ± 0.3 b	638.6 ± 0.7 c	1137.5 ± 2.6 a
Arg	1024.9 ± 1.2 c	1688.7 ± 2.2 a	644.0 ± 1.0 d	1452.3 ± 3.1 b
Pro	1326.3 ± 5.2 d	2031.9 ± 0.9 b	1654.3 ± 4.4 c	2489.8 ± 1.7 a
Total FAA (17)	25,054.8 ± 28.0 d	38,632.3 ± 49.5 b	27,769.4 ± 33.8 c	44,524.7 ± 31.2 a
Gln	994.5 ± 7.9 c	1650.0 ± 25.5 a	995.4 ± 8.7 c	1189.0 ± 12.0 b
Tau	3362.9 ± 5.2 b	3089.7 ± 1.6 d	3238.4 ± 3.4 c	3915.6 ± 4.1 a

**Note:** Values are presented as mean ± SD (*n* = 3 tank-level pooled biological replicates). Different lowercase letters within a row indicate significant differences among the four treatment combinations based on Tukey’s post hoc test following two-way ANOVA (*p* < 0.05). FW, freshwater control; S, salinity stress; A, alkalinity stress; SA, combined saline–alkaline stress. The full two-way ANOVA outputs for liver FAA contents are provided in Supplementary Table S3.

**Table 5:** Free amino acid contents in brain of 10 cm mandarin fish at 96 h under different treatments (µg/g sample).

Amino Acid	FW	S	A	SA
Asp	641.7 ± 1.5 b	486.4 ± 0.3 c	486.2 ± 18.0 c	707.4 ± 3.7 a
Thr	383.1 ± 0.3 b	328.6 ± 3.1 c	333.3 ± 13.8 c	434.8 ± 1.6 a
Ser	472.8 ± 2.1 b	378.8 ± 3.8 c	387.3 ± 14.7 c	607.4 ± 1.8 a
Glu	1375.4 ± 9.4 a	1349.5 ± 1.4 a	1002.3 ± 27.8 c	1164.7 ± 4.8 b
Gly	456.4 ± 0.6 b	381.0 ± 0.8 d	424.9 ± 16.1 c	580.2 ± 1.0 a
Ala	482.8 ± 1.7 b	382.9 ± 0.8 c	336.3 ± 12.9 d	512.0 ± 0.6 a
Cystine	7.9 ± 0.3 b	4.7 ± 0.2 d	6.5 ± 0.1 c	13.4 ± 0.5 a
Val	188.8 ± 2.5 b	130.0 ± 0.8 d	141.2 ± 8.0 c	233.5 ± 0.3 a
Met	159.8 ± 1.9 b	110.4 ± 0.5 d	124.1 ± 2.4 c	188.3 ± 2.5 a
Ile	161.1 ± 1.4 b	105.3 ± 0.6 d	120.7 ± 2.8 c	181.7 ± 1.4 a
Leu	373.8 ± 0.7 b	184.5 ± 0.1 d	305.5 ± 10.6 c	389.8 ± 0.7 a
Tyr	186.3 ± 4.2 b	123.2 ± 0.9 d	137.1 ± 2.4 c	211.3 ± 1.9 a
Phe	2205.1 ± 8.3 a	1688.1 ± 6.3 c	2002.3 ± 125.2 b	2102.3 ± 6.9 ab
Lys	79.6 ± 0.7 a	58.7 ± 1.2 b	16.2 ± 0.6 d	24.8 ± 1.0 c
His	1881.2 ± 2.0 c	2036.6 ± 3.3 b	1606.8 ± 70.7 d	2195.2 ± 3.3 a
Arg	30.5 ± 0.3 c	43.0 ± 1.5 b	13.1 ± 0.3 d	47.8 ± 1.3 a
Pro	262.4 ± 2.6 b	176.6 ± 4.8 c	177.4 ± 3.8 c	297.3 ± 8.6 a
Total FAA (17)	9348.7 ± 14.4 b	7968.3 ± 13.0 c	7621.2 ± 320.9 c	9891.9 ± 10.7 a
Gln	775.3 ± 9.6 d	1402.4 ± 14.6 b	1039.2 ± 46.0 c	2223.4 ± 5.4 a
Tau	3797.9 ± 1.9 c	4255.5 ± 19.9 b	3496.8 ± 120.3 d	4523.3 ± 4.1 a

**Note:** Values are presented as mean ± SD (*n* = 3 tank-level pooled biological replicates). Different lowercase letters within a row indicate significant differences among the four treatment combinations based on Tukey’s post hoc test following two-way ANOVA (*p* < 0.05). FW, freshwater control; S, salinity stress; A, alkalinity stress; SA, combined saline–alkaline stress. The full two-way ANOVA outputs for brain FAA contents are provided in Supplementary Table S4.

**Table 6:** Free amino acid contents in kidney of 10 cm mandarin fish at 96 h under different treatments (µg/g sample).

Amino Acid	FW	S	A	SA
Asp	2211.0 ± 3.9 d	2423.6 ± 2.8 c	3075.4 ± 10.0 a	2971.9 ± 14.5 b
Thr	1639.8 ± 2.4 d	1809.5 ± 4.3 c	2269.8 ± 3.9 a	2038.3 ± 0.3 b
Ser	1811.6 ± 3.4 d	2083.9 ± 3.5 c	2470.7 ± 1.6 a	2283.5 ± 0.2 b
Glu	3313.7 ± 9.9 d	3561.2 ± 10.4 c	4030.3 ± 20.5 a	3653.2 ± 5.7 b
Gly	1126.5 ± 3.3 d	1276.2 ± 0.9 c	1483.4 ± 1.1 a	1462.4 ± 0.6 b
Ala	2818.9 ± 6.9 d	2971.5 ± 0.7 c	3660.3 ± 2.4 a	3191.8 ± 3.5 b
Cystine	5.1 ± 0.1 c	3.2 ± 0.1 d	10.9 ± 0.3 b	26.8 ± 0.2 a
Val	1900.5 ± 4.9 d	2073.8 ± 1.8 c	2889.2 ± 3.4 a	2469.9 ± 3.4 b
Met	836.1 ± 2.6 c	809.4 ± 1.2 d	1403.6 ± 1.5 a	1181.4 ± 0.5 b
Ile	1428.9 ± 2.8 d	1597.8 ± 1.0 c	2378.2 ± 2.1 a	2036.4 ± 4.0 b
Leu	2671.9 ± 5.3 d	3132.3 ± 2.7 c	4286.1 ± 3.8 a	3908.8 ± 1.4 b
Tyr	1233.4 ± 2.3 d	1536.4 ± 2.7 c	2112.9 ± 10.7 a	2034.9 ± 3.1 b
Phe	1669.9 ± 1.7 d	2182.9 ± 8.2 c	2653.8 ± 11.3 a	2602.5 ± 13.9 b
Lys	1801.3 ± 5.6 b	984.4 ± 6.0 c	2365.9 ± 9.4 a	917.6 ± 8.4 d
His	574.8 ± 1.4 d	581.7 ± 1.1 c	1153.3 ± 2.2 a	962.5 ± 0.7 b
Arg	441.9 ± 1.8 d	624.2 ± 2.1 c	1340.4 ± 3.9 a	1138.4 ± 3.2 b
Pro	1639.0 ± 12.8 d	2031.1 ± 1.4 b	2105.2 ± 5.1 a	1973.3 ± 5.1 c
Total FAA (17)	27,124.4 ± 35.9 d	29,683.2 ± 27.6 c	39,689.4 ± 42.7 a	34,853.6 ± 17.6 b
Gln	686.5 ± 12.6 c	924.7 ± 6.8 b	991.6 ± 30.0 a	895.3 ± 13.2 b
Tau	2990.2 ± 7.6 d	3855.5 ± 5.0 a	3070.3 ± 4.5 c	3213.7 ± 6.0 b

**Note:** Values are presented as mean ± SD (*n* = 3 tank-level pooled biological replicates). Different lowercase letters within a row indicate significant differences among the four treatment combinations based on Tukey’s post hoc test following two-way ANOVA (*p* < 0.05). FW, freshwater control; S, salinity stress; A, alkalinity stress; SA, combined saline–alkaline stress. The full two-way ANOVA outputs for kidney FAA contents are provided in Supplementary Table S5.

## Data Availability

The data presented in this study are available within the article.

## Supplementary Materials

The following supporting information can be downloaded at: https://www.mdpi.com/article/10.3390/life16061031/s1, Table S1: Two-way ANOVA outputs for FAA contents in plasma of mandarin fish; Table S2: Two-way ANOVA outputs for FAA contents in muscle of mandarin fish; Table S3: Two-way ANOVA outputs for FAA contents in liver of mandarin fish; Table S4: Two-way ANOVA outputs for FAA contents in brain of mandarin fish; Table S5: Two-way ANOVA outputs for FAA contents in kidney of mandarin fish.
