# Supplementary material for: Tissue-Specific Redistribution of Free Amino Acids in Mandarin Fish (Siniperca chuatsi) Under Acute Salinity, Alkalinity and Combined Saline–Alkaline Stress
Source: Life (Basel). 2026 Jun 19;16(6):1031. doi: 10.3390/life16061031 (PMC13302728; doi:10.3390/life16061031)
Supplement: Supplementary file 1 [file life-16-01031-s001.zip › life-4335876-supplementary.pdf]

Supplementary Tables S1–S5. Two-way ANOVA outputs for FAA contents in plasma, muscle, liver, brain, and kidney of mandarin fish under salinity, alkalinity, and combined saline-alkaline stress.

Note: For each FAA-related component within each tissue, two-way ANOVA was performed using salinity and alkalinity as fixed factors. df indicates the numerator and denominator degrees of freedom, respectively. S × A represents the salinity × alkalinity interaction. F, F-value; P, P-value. F-values are rounded to two decimal places, and P-values are shown to four decimal places or as <0.0001. P < 0.05 was considered statistically significant. FAA, free amino acid; Total FAA (17), the sum of 17 conventional free amino acids.

Supplementary Table S1. Two-way ANOVA outputs for FAA contents in plasma of mandarin fish.

FAA	Salinity			Alkalinity			S × A		
	df	F	*P*	df	F	*P*	df	F	*P*
Asp	1, 8	310.36	<0.0001	1, 8	711.43	<0.0001	1, 8	48.58	0.0001
Thr	1, 8	0.12	0.7413	1, 8	634.30	<0.0001	1, 8	300.46	<0.0001
Ser	1, 8	3.61	0.0942	1, 8	1664.79	<0.0001	1, 8	110.32	<0.0001
Glu	1, 8	2.48	0.1537	1, 8	235.25	<0.0001	1, 8	0.04	0.8388
Gly	1, 8	2.42	0.1584	1, 8	1951.06	<0.0001	1, 8	231.52	<0.0001
Ala	1, 8	820.68	<0.0001	1, 8	6265.14	<0.0001	1, 8	20.68	0.0019
Cystine	1, 8	96.33	<0.0001	1, 8	208.33	<0.0001	1, 8	16.33	0.0037
Val	1, 8	926.32	<0.0001	1, 8	379.57	<0.0001	1, 8	53.50	<0.0001
Met	1, 8	0.77	0.4068	1, 8	678.15	<0.0001	1, 8	9.16	0.0164
Ile	1, 8	4581.32	<0.0001	1, 8	2324.76	<0.0001	1, 8	552.61	<0.0001
Leu	1, 8	8339.98	<0.0001	1, 8	4340.77	<0.0001	1, 8	693.60	<0.0001
Tyr	1, 8	406.40	<0.0001	1, 8	135.13	<0.0001	1, 8	137.10	<0.0001
Phe	1, 8	4.56	0.0652	1, 8	1.60	0.2420	1, 8	78.24	<0.0001
Lys	1, 8	10133.77	<0.0001	1, 8	6075.06	<0.0001	1, 8	15.57	0.0043
His	1, 8	86.88	<0.0001	1, 8	332.36	<0.0001	1, 8	1.71	0.2274
Arg	1, 8	307.63	<0.0001	1, 8	40.75	0.0002	1, 8	10.66	0.0114
Pro	1, 8	406.56	<0.0001	1, 8	60.74	<0.0001	1, 8	28.65	0.0007
Total FAA (17)	1, 8	6966.52	<0.0001	1, 8	11097.81	<0.0001	1, 8	21.88	0.0016
Gln	1, 8	49.11	0.0001	1, 8	1067.83	<0.0001	1, 8	39.36	0.0002
Tau	1, 8	7967.78	<0.0001	1, 8	788.03	<0.0001	1, 8	252.11	<0.0001

Supplementary Table S2. Two-way ANOVA outputs for FAA contents in muscle of mandarin fish.

FAA	Salinity			Alkalinity			S × A		
	df	F	*P*	df	F	*P*	df	F	*P*
Asp	1, 8	429.23	<0.0001	1, 8	594.68	<0.0001	1, 8	2605.96	<0.0001
Thr	1, 8	22276.81	<0.0001	1, 8	1297.10	<0.0001	1, 8	475.42	<0.0001
Ser	1, 8	51752.01	<0.0001	1, 8	1329.62	<0.0001	1, 8	390.20	<0.0001
Glu	1, 8	81928.89	<0.0001	1, 8	5171.23	<0.0001	1, 8	10404.88	<0.0001
Gly	1, 8	582.50	<0.0001	1, 8	3351.08	<0.0001	1, 8	14979.67	<0.0001
Ala	1, 8	4408.49	<0.0001	1, 8	0.03	0.8595	1, 8	581.83	<0.0001
Cystine	1, 8	4.00	0.0805	1, 8	544.44	<0.0001	1, 8	544.44	<0.0001
Val	1, 8	666.67	<0.0001	1, 8	67.13	<0.0001	1, 8	0.35	0.5686
Met	1, 8	4.16	0.0756	1, 8	15.89	0.0040	1, 8	0.28	0.6081
Ile	1, 8	13599.22	<0.0001	1, 8	21.51	0.0017	1, 8	5.90	0.0412
Leu	1, 8	2789.79	<0.0001	1, 8	3.61	0.0940	1, 8	12.66	0.0074
Tyr	1, 8	25.21	0.0010	1, 8	45.82	0.0001	1, 8	6.73	0.0319
Phe	1, 8	12.76	0.0073	1, 8	3.68	0.0912	1, 8	92.09	<0.0001
Lys	1, 8	15284.12	<0.0001	1, 8	17802.78	<0.0001	1, 8	13572.96	<0.0001
His	1, 8	8790.45	<0.0001	1, 8	1.38	0.2739	1, 8	1577.65	<0.0001
Arg	1, 8	18.59	0.0026	1, 8	187.35	<0.0001	1, 8	178.10	<0.0001
Pro	1, 8	2011.29	<0.0001	1, 8	27.14	0.0008	1, 8	395.81	<0.0001
Total FAA (17)	1, 8	39377.47	<0.0001	1, 8	1750.83	<0.0001	1, 8	14019.27	<0.0001
Gln	1, 8	2356.45	<0.0001	1, 8	236.45	<0.0001	1, 8	184.09	<0.0001
Tau	1, 8	73.15	<0.0001	1, 8	7.99	0.0223	1, 8	330.22	<0.0001

Supplementary Table S3. Two-way ANOVA outputs for FAA contents in liver of mandarin fish.

FAA	Salinity			Alkalinity			S × A		
	df	F	*P*	df	F	*P*	df	F	*P*
Asp	1, 8	221762.88	<0.0001	1, 8	37911.34	<0.0001	1, 8	961.85	<0.0001
Thr	1, 8	240148.66	<0.0001	1, 8	35780.25	<0.0001	1, 8	365.70	<0.0001
Ser	1, 8	593680.24	<0.0001	1, 8	182271.13	<0.0001	1, 8	790.44	<0.0001
Glu	1, 8	226326.32	<0.0001	1, 8	41922.74	<0.0001	1, 8	1184.79	<0.0001
Gly	1, 8	381375.05	<0.0001	1, 8	70775.75	<0.0001	1, 8	11.38	0.0097
Ala	1, 8	287206.49	<0.0001	1, 8	118660.01	<0.0001	1, 8	4140.98	<0.0001
Cystine	1, 8	1204.17	<0.0001	1, 8	1380.17	<0.0001	1, 8	194.94	<0.0001
Val	1, 8	235036.19	<0.0001	1, 8	28040.91	<0.0001	1, 8	35.25	0.0003
Met	1, 8	1962185.13	<0.0001	1, 8	149389.98	<0.0001	1, 8	33840.54	<0.0001
Ile	1, 8	543095.46	<0.0001	1, 8	34739.90	<0.0001	1, 8	2167.59	<0.0001
Leu	1, 8	1178619.48	<0.0001	1, 8	143635.69	<0.0001	1, 8	21209.54	<0.0001
Tyr	1, 8	78349.84	<0.0001	1, 8	3278.53	<0.0001	1, 8	0.11	0.7498
Phe	1, 8	19400.71	<0.0001	1, 8	5115.91	<0.0001	1, 8	356.59	<0.0001
Lys	1, 8	7746.67	<0.0001	1, 8	7147.49	<0.0001	1, 8	3086.43	<0.0001
His	1, 8	88901.73	<0.0001	1, 8	276.39	<0.0001	1, 8	317.85	<0.0001
Arg	1, 8	385542.38	<0.0001	1, 8	67798.06	<0.0001	1, 8	3716.33	<0.0001
Pro	1, 8	142253.25	<0.0001	1, 8	37002.27	<0.0001	1, 8	1011.34	<0.0001
Total FAA (17)	1, 8	515660.04	<0.0001	1, 8	41518.95	<0.0001	1, 8	5659.33	<0.0001
Gln	1, 8	2328.07	<0.0001	1, 8	683.22	<0.0001	1, 8	688.97	<0.0001
Tau	1, 8	8410.79	<0.0001	1, 8	25356.42	<0.0001	1, 8	46546.47	<0.0001

Supplementary Table S4. Two-way ANOVA outputs for FAA contents in brain of mandarin fish.

FAA	Salinity			Alkalinity			S × A		
	df	F	*P*	df	F	*P*	df	F	*P*
Asp	1, 8	38.34	0.0003	1, 8	37.95	0.0003	1, 8	1251.47	<0.0001
Thr	1, 8	32.51	0.0005	1, 8	46.93	0.0001	1, 8	358.50	<0.0001
Ser	1, 8	202.03	<0.0001	1, 8	260.14	<0.0001	1, 8	1251.63	<0.0001
Glu	1, 8	63.24	<0.0001	1, 8	1057.32	<0.0001	1, 8	120.45	<0.0001
Gly	1, 8	73.07	<0.0001	1, 8	321.73	<0.0001	1, 8	608.80	<0.0001
Ala	1, 8	101.92	<0.0001	1, 8	5.37	0.0491	1, 8	1347.34	<0.0001
Cystine	1, 8	117.15	<0.0001	1, 8	448.08	<0.0001	1, 8	831.20	<0.0001
Val	1, 8	46.82	0.0001	1, 8	131.42	<0.0001	1, 8	960.19	<0.0001
Met	1, 8	41.55	0.0002	1, 8	334.81	<0.0001	1, 8	2429.10	<0.0001
Ile	1, 8	6.58	0.0334	1, 8	316.33	<0.0001	1, 8	3321.27	<0.0001
Leu	1, 8	291.66	<0.0001	1, 8	495.97	<0.0001	1, 8	1978.58	<0.0001
Tyr	1, 8	13.16	0.0067	1, 8	161.02	<0.0001	1, 8	2012.83	<0.0001
Phe	1, 8	32.94	0.0004	1, 8	8.47	0.0196	1, 8	72.14	<0.0001
Lys	1, 8	137.26	<0.0001	1, 8	8589.28	<0.0001	1, 8	793.11	<0.0001
His	1, 8	330.66	<0.0001	1, 8	8.01	0.0221	1, 8	112.06	<0.0001
Arg	1, 8	1643.27	<0.0001	1, 8	118.22	<0.0001	1, 8	363.97	<0.0001
Pro	1, 8	29.44	0.0006	1, 8	32.15	0.0005	1, 8	1073.12	<0.0001
Total FAA (17)	1, 8	22.97	0.0014	1, 8	1.12	0.3218	1, 8	386.41	<0.0001
Gln	1, 8	4008.55	<0.0001	1, 8	1438.05	<0.0001	1, 8	379.08	<0.0001
Tau	1, 8	444.07	<0.0001	1, 8	0.22	0.6490	1, 8	65.28	<0.0001

Supplementary Table S5. Two-way ANOVA outputs for FAA contents in kidney of mandarin fish.

FAA	Salinity			Alkalinity			S × A		
	df	F	*P*	df	F	*P*	df	F	*P*
Asp	1, 8	106.86	<0.0001	1, 8	17883.03	<0.0001	1, 8	895.20	<0.0001
Thr	1, 8	288.45	<0.0001	1, 8	55463.21	<0.0001	1, 8	12104.39	<0.0001
Ser	1, 8	822.79	<0.0001	1, 8	83656.69	<0.0001	1, 8	23950.87	<0.0001
Glu	1, 8	76.32	<0.0001	1, 8	2972.70	<0.0001	1, 8	1773.77	<0.0001
Gly	1, 8	3870.02	<0.0001	1, 8	68915.33	<0.0001	1, 8	6808.06	<0.0001
Ala	1, 8	4466.89	<0.0001	1, 8	50470.63	<0.0001	1, 8	17271.08	<0.0001
Cystine	1, 8	3328.30	<0.0001	1, 8	14611.32	<0.0001	1, 8	5380.30	<0.0001
Val	1, 8	3611.22	<0.0001	1, 8	114434.69	<0.0001	1, 8	20946.50	<0.0001
Met	1, 8	17220.00	<0.0001	1, 8	245358.39	<0.0001	1, 8	10630.20	<0.0001
Ile	1, 8	3059.89	<0.0001	1, 8	197318.43	<0.0001	1, 8	26723.69	<0.0001
Leu	1, 8	402.74	<0.0001	1, 8	333867.84	<0.0001	1, 8	40992.22	<0.0001
Tyr	1, 8	1115.36	<0.0001	1, 8	41831.83	<0.0001	1, 8	3198.16	<0.0001
Phe	1, 8	1639.42	<0.0001	1, 8	15146.50	<0.0001	1, 8	2448.95	<0.0001
Lys	1, 8	67852.17	<0.0001	1, 8	3276.49	<0.0001	1, 8	5271.19	<0.0001
His	1, 8	12063.52	<0.0001	1, 8	328164.98	<0.0001	1, 8	13941.61	<0.0001
Arg	1, 8	35.25	0.0003	1, 8	180654.23	<0.0001	1, 8	13367.01	<0.0001
Pro	1, 8	925.91	<0.0001	1, 8	2281.55	<0.0001	1, 8	3754.59	<0.0001
Total FAA (17)	1, 8	3723.64	<0.0001	1, 8	225911.48	<0.0001	1, 8	39271.74	<0.0001
Gln	1, 8	47.17	0.0001	1, 8	178.06	<0.0001	1, 8	262.22	<0.0001
Tau	1, 8	22010.71	<0.0001	1, 8	6824.90	<0.0001	1, 8	11271.26	<0.0001
